# A Competitive Study Using Electrospinning and Phase Inversion to Prepare Polymeric Membranes for Oil Removal

**DOI:** 10.3390/membranes13050474

**Published:** 2023-04-28

**Authors:** Thamer Diwan, Zaidun N. Abudi, Mustafa H. Al-Furaiji, Arian Nijmeijer

**Affiliations:** 1 Environmental Engineering Department, College of Engineering, Mustansiriyah University, Baghdad 10052, Iraq or thamerdiwanfdzm@uomustansiriyah.edu.iq (T.D.); zaidun.naji77@uomustansiriyah.edu.iq (Z.N.A.); 2 Technical Directorate, Ministry of Environment, Baghdad 10066, Iraq; 3 Environment and Water Directorate, Ministry of Science and Technology, Baghdad 10066, Iraq; 4 Inorganic Membranes, Department of Science and Technology, MESA+ Institute for Nanotechnology, University of Twente, P.O. Box 217, 7500 AE Enschede, The Netherlands

**Keywords:** electrospinning, oil removal, PAN polymer, phase inversion, DMF

## Abstract

Polyacrylonitrile (PAN) is a popular polymer that can be made into membranes using various techniques, such as electrospinning and phase inversion. Electrospinning is a novel technique that produces nonwoven nanofiber-based membranes with highly tunable properties. In this research, electrospun PAN nanofiber membranes with various concentrations (10, 12, and 14% PAN/dimethylformamide (DMF)) were prepared and compared to PAN cast membranes prepared by the phase inversion technique. All of the prepared membranes were tested for oil removal in a cross-flow filtration system. A comparison between these membranes’ surface morphology, topography, wettability, and porosity was presented and analyzed. The results showed that increasing the concentration of the PAN precursor solution increases surface roughness, hydrophilicity, and porosity and, consequently, enhances the membrane performance. However, the PAN cast membranes showed a lower water flux when the precursor solution concentration increased. In general, the electrospun PAN membranes performed better in terms of water flux and oil rejection than the cast PAN membranes. The electrospun 14% PAN/DMF membrane gave a water flux of 250 LMH and a rejection of 97% compared to the cast 14% PAN/DMF membrane, which showed a water flux of 117 LMH and 94% oil rejection. This is mainly because the nanofibrous membrane showed higher porosity, higher hydrophilicity, and higher surface roughness compared to the cast PAN membranes at the same polymer concentration. The porosity of the electrospun PAN membrane was 96%, while it was 58% for the cast 14% PAN/DMF membrane.

## 1. Introduction

In recent decades, oily wastewater treatment has become a research hotspot due to its significant environmental damage [1]. It has a harmful effect on human health, ecological systems, and some industrial activities [2]. 

In many countries, especially in oil-producing countries such as Iraq, water shortages are still a large problem [3]. The recovery, recycling, and treatment of wastewater are essential for the protection and sustainable exploitation of water resources. Solids, organics, inorganics, toxins, emulsions, and other complex pollutants can potentially be present in various industrial wastewater streams [4]. It has been reported that oil fields and refineries are the largest sources of oily wastewater [5]. The concentration and type of oil in the wastewater mainly depend on the emulsification efficiency and the crude oil’s nature [6]. For instance, in the Rumaila oil field, processing wastewater is routed to numerous unlined disposal pits and/or injected into the subsurface in the Dammam Formation via injection wells without being treated [7]. At the same time, this oil field requires a large amount of injection water (more than one million barrels/day) to sustain oil production [8]. Therefore, the need for more water to increase oil production can be supplied partially or primarily by treating the oily wastewater and reusing it for reinjection [9].

Oil in water can be categorized as free, dispersed, and emulsified. The first two types—when existing in wastewater—can be removed by simple physical processes such as separation by gravity, skimming [10], air flotation [11], mechanical extraction [12], coagulation [13], chemical degradation [14], and flocculation [15]. These techniques have different disadvantages, including their high cost, the use of chemical compounds, corrosion, a low efficiency, recontamination problems, and the need for extensive land areas [16,17]. Emulsified oil is usually very difficult to deal with using traditional methods [18]. This is primarily because of the droplet sizes for emulsified oils, which are usually smaller than 10 µm [19] and lead to complex treatment processes [20]. Therefore, many researchers have tried to discover effective methods to treat emulsified oily wastewater [21,22].

Membrane processes are one of the promising technologies that can be used in treating polluted water streams due to their low capital cost, high-performance efficiency [23], small carbon footprint [17], energy efficiency, and the ability to generate a high-quality permeate. In the past decades, membrane technology has been considered a suitable and applicable method to treat water by removing a wide range of contaminants, especially in oily wastewater [24]. A significant challenge in the practical implementation of membrane filtration is membrane fouling. The main factor responsible for this is the formation of a filter cake composed of oil droplets that accumulate on the membrane surface during filtration [25]. Moreover, removing the fouling from the membrane surface is possible by employing backwashing, ultrasonic vibration, or periodic flow. By doing so, the lifespan of the membrane can be extended [26].

In general, the membrane processes are divided into four kinds depending on the pressure applied on the membrane, including microfiltration (MF) [27], ultrafiltration (UF) [28], nanofiltration (NF) [29], and reverse osmosis (RO) [30], in which the applied pressure increases from 1 bar for MF to about 80 bar for RO [31]. Oil removal is typically conducted using polymeric membranes or ceramic membranes. A wide range of polymeric materials can be used to prepare UF membranes, such as [32] polyacrylonitrile (PAN) [33], polyvinylidene difluoride (PVDF) [34], polyamide (PA) [35], polyether sulfone (PES) [36], polysulfone (PSF) [37], nylon (NY) [38], cellulose acetate (CA) [39], polyvinyl alcohol (PVA) [40], polystyrene (PS) [41], polyurethane (PU) [42], etc.

Two main methods for fabricating polymeric membranes are phase inversion and electrospinning. In the phase inversion technique, a homogenous polymeric solution is transformed in a controlled process from a liquid phase into a solid phase by immersing the polymeric solution in a water bath [43]. At the phase inversion technique, the polymeric membranes are formed as thin film sheets or hollow fibers [32].

The electrospinning process includes stretching the polymeric solution in an electrostatic field, where the volatile solvent is removed by evaporation, and the nanofibers are deposited on a rotating drum [44]. The electrospinning process has the ability to produce fibers with highly tunable properties in which the diameter of the electrospun fibers ranges from 10 nm to 100 μm [45,46].

Zhang et al. 2020 developed a composite membrane by incorporating s-kaolin particles into an electrospun PAN membrane. They showed that this modified membrane effectively separated different oil-in-water emulsions under applied pressure [47]. In another exciting study in this field, a new type of thin film composite (TFC) membrane for oil removal was developed and tested [48]. The developed TFC membrane consisted of a nonwoven polyester (PET) support, an electrospun polyacrylonitrile (PAN) nanofibrous mid-layer, and a polysulfone (PSF) composite coating top layer. The researchers compared this membrane to an asymmetric ultrafiltration (UF) membrane that was fabricated using the phase inversion method. The results showed that the pure water flux of the TFC membrane increased from 20 to 160% compared to that of the PSF asymmetric membrane.

Another group of researchers developed a membrane by incorporating polyacrylonitrile fibers with graphene oxide. The resulting membrane displayed remarkable characteristics, including superhydrophilicity, low oil adhesion, and high flux. Moreover, it exhibited an oil rejection of at least 98% and excellent antifouling properties while separating oil–water emulsions [49]. In a different approach, needleless electrospinning was used to produce PAN membranes with a hollow fiber structure [50]. The relationship between the crystal structure and its tendency to form hollow fiber PAN membranes was reported in this research.

Recently, Alkarbouly and Waisi (2022) have fabricated electrospun nanofibers with varying diameters (from 150 to 400 nm) and porosity (from 91% to 96%) using different concentrations (i.e., 8%, 11%, and 14%) of a PAN/DMF precursor solution. These nanofibers have demonstrated a water flux of up to 120 LMH and an oil removal of about 92.5%. Additionally, they prepared dual-layer nanofiber membranes consisting of PAN-PMMA to achieve a high flux and minimize fouling [51].

In this study, a detailed comparison was performed between electrospinning and phase inversion to prepare PAN membranes with different precursor solution concentrations (10, 12, and 14 wt. %. The obtained membranes were characterized and assessed using various analytical techniques such as SEM, AFM, contact angles, and porosity measurement. The performance of the prepared membranes was evaluated in a cross-flow filtration system for oil removal.

## 2. Materials and Methods

### 2.1. Materials

Polyacrylonitrile (PAN) (Mw = 150,000 g/mol) was purchased from Shanghai Macklin Biochemical Co., Ltd. (Shanghai, China). *N*,*N*-dimethylformamide (DMF) (Mw = 73.1 g/mol) was ordered from Thomas Baker (Chemicals) Pvt. Ltd. (Mumbai, India). Tween 80 was purchased from Hopkin & Williams Ltd. (Wolverhampton, UK), while kerosene (Mw = 175 g/mol) was provided by the midland Iraqi refineries company.

### 2.2. Polymeric Solutions Preparation

Different concentrations (i.e., 10, 12, and 14 wt. %) of polyacrylonitrile polymeric solution were prepared by dissolving the proper amount of PAN powder in DMF solvent. The precursor solution was continuously stirred using a magnetic stirrer for 6 h at 60 °C. The formed polymeric solution became clear and homogenous and was left for 18 h at room temperature to remove trapped air bubbles.

### 2.3. Preparation of the Electrospun Nanofibers Membranes

All the nonwoven nanofiber membranes were fabricated using the electrospinning process. The homemade electrospinning setup consisted of a syringe pump, a high-voltage power supply, and a rotating drum. The PAN/DMF solution was placed in a disposable plastic syringe (5 mL) that had a small inner diameter with a capillary metal needle (gauge 23 G). The polymeric solution was pumped at a flow rate of 1 mL/h with a high voltage of 30 kV. The distance between the needle tip and the collecting drum was fixed at 17 cm. The electrospinning process was conducted at room temperature and with ambient humidity.

**Figure 1:**
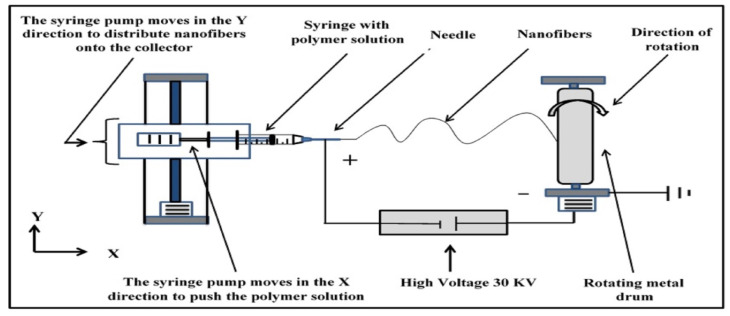
Schematic diagram of the electrospinning.

An electrostatic force formed between the needle tip (positive side) and the rotating metal drum collector with a speed of 70 rpm (negative side), forcing the flow of the polymeric solution through the air and forming the nanofibers. At the same time, the solvent evaporated, resulting in the formation of a mat of nonwoven nanofibers on the surface of the collector with dimensions of 25 cm × 25 cm. A schematic diagram of the electrospinning system is shown in Figure 1.

### 2.4. Preparation of the Cast Membranes

The flat sheet cast PAN membranes were prepared via phase inversion using polymeric solutions of PAN in DMF at various concentrations (10, 12, and 14 wt. %). The cast solution was poured on clean glass before a casting knife (Film applicator KTQ-II, Xiamen TOB New Energy Technology Co., Ltd., Fujian Province, China) was used to create a thin film layer with a thickness of 180 microns. Then, the glass with the thin film of cast solution was immersed in DI water, resulting in the immediate formation of the PAN membrane. Later, the PAN membrane was dried in an oven at 70 °C for 10 min to remove the remaining solvent. Prior to testing, the obtained membrane was stored in DI water at 4 °C for 24 h [52].

### 2.5. Membrane Characterization

To determine the morphology of the surface structure and cross-section before and after tests, scanning electron microscopy (SEM) images of membrane samples were obtained using field emission SEM (FESEM, Inspect F50, FEI Technologies Inc., Hillsboro, OR, USA). Imaging was conducted using an accelerating voltage of 20 kV. Before SEM analysis, all samples were sputtered with a thin layer of gold to provide a conducting face.

The average diameter of the PAN nanofibers was estimated by analyzing the SEM images using Image J 1.48v software (National Institutes of Health, Bethesda, MD, USA) [53]. At least thirty nanofibers were considered for each nanofiber membrane sample, and the average value was recorded.

The topography of the prepared membranes was characterized using an atomic force microscope (AFM, XE 100) manufactured by Park Systems, Suwon, Republic of Korea. The samples were scanned using the tapping mode three times. Using arithmetic means, the absolute height of the surface roughness (Ra) was calculated from the roughness analysis report provided by AFM.

The Dynamic Mechanical Analyzer (DMA) (AG-A10T, Shimadzu, Kyoto, Japan) was used to evaluate the mechanical strength of the membranes. The specimen size of 10 cm in length and 1 cm in width was used for the tests, which were all performed at 25 °C and with ambient humidity.

The water contact angle of the PAN membranes was examined to indicate the wettability using a Contact Angle Measuring Instrument (Theta Lite TL-101, Biolin Scientific, Bangkok, Thailand). Three different places for each membrane sample were measured, and the average value was recorded.

The porosity of all membrane samples was estimated using the gravimetrical method, in which the membrane sample was cut into disks with a radius of 1.5 cm. The dry sample was weighed before (W_dry_) and after (W_wet_) immersion in isopropyl alcohol (IPA). The following equation was used to calculate the membrane porosity (ε) [54].

(1) $$\varepsilon =\frac{\frac{W_{wet}\;-\;W_{dry}}{\varrho_{IPA}}}{V}\times 100\%$$

where ρ_IPA_ is the density of IPA, and V is the total volume of the sample. Each membrane was tested at least three times.

The oil droplet size of the prepared emulsion was analyzed by Labor scope (LOMO Laboroscope AL-200, Northbrook, IL, USA). The measurement was performed again after 4 h of emulsified oil solution preparation (1000 mg/L). The oil concentration in water was measured using a UV-Vis spectrophotometer (Model Vis-722G & UV-9200/Biotech Engineering Management Co. Ltd., Nicosia, Cyprus) using a 1.0 cm quartz cell at a wavelength of 290 nm.

### 2.6. Oil Removal Performance Test

To prepare a stable emulsion solution of a 1000 mg/L concentration of oil, 1 g of kerosene and 0.1 g of Tween 80 were added to 1000 mL of distilled water. A homogenizer device (SRH-S 450 Lab High-Shear Emulsifier, Shanghai, China) was used to create an emulsion by extreme shear stirring at 10,000 rpm for 10 min at room temperature [19].

**Figure 2:**
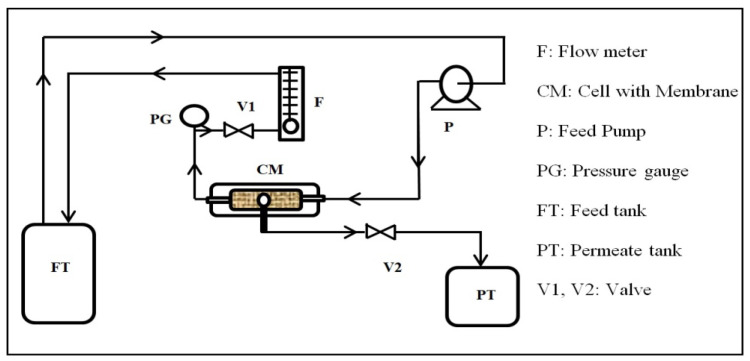
Schematic diagram of the cross-flow filtration system.

The oil removal experiments were carried out using a cross-flow filtration system, as shown in Figure 2. The testing setup consisted of a feed tank, a feed pump, a permeate tank, a flow meter, a valve, a pressure gauge, and a homemade cross-flow filtration cell. The cell has a rectangular shape with dimensions of 6 × 12 cm^2^. The membrane piece was placed into the cell, which was reinforced on a thin net-kind mesh fitted using a rectangular rubber gasket. The effective area of the membrane piece was 2.5 × 8 cm^2^. The feed solution was circulated through the cell by the feed pump of the feed tank.

The permeate was collected in a beaker, and the flux was obtained by measuring the time to collect a certain volume of water. The flow rate and pressure were adjusted by controlling the valves and recorded. The flow and pressure were displayed by the flow meter and the pressure gauge, respectively.

The separation efficiency of the oil emulsions and the water flux of the emulsions were determined by calculating the quantity of permeate per unit of time according to Equations (2) and (3), respectively [5]:

(2) $$R\%=\left[1-\left(\frac{C_{t}}{C_{o}}\right)\right]\times 100\%$$

(3) $$J=\frac{V}{A\times t}$$

where R (%) is the oil rejection percentage and C**_t_** and C**_o_** (mg/L) are the oil concentration of the collected water and the feed, respectively. J is the water flux of the membrane (LMH), A (m^2^) is the active area of the membrane, V (L) is the volume of the permeate, and t (h) is the experiment time. Three samples were tested for each experiment to obtain the average value.

## 3. Results and Discussion

### 3.1. Characterizing the Solution of Emulsified Oil

**Figure 3:**
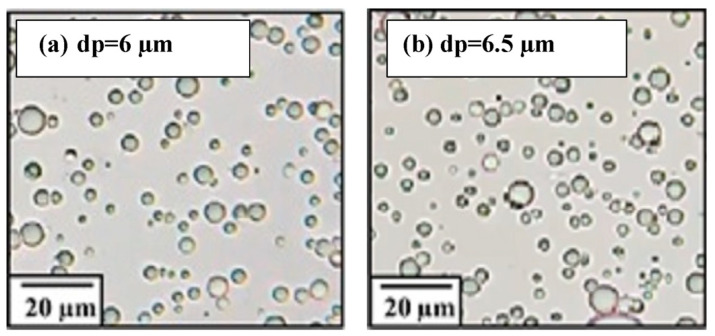
The size of oil droplets before and after the oil removal experiment depicted in digital images after different time intervals: (**a**) 1 h; (**b**) 4 h.

Oil can exist in water in three different forms: free or floating oil (with droplets larger than 150 µm), dispersed oil (with droplet sizes ranging from 150 to 20 µm), and emulsified oil (with droplets smaller than 20 µm) [55]. As shown in Figure 3, the oil droplet size was smaller than 20 µm before and after the testing experiment; this confirms that the prepared samples were in the emulsified form.

### 3.2. Membrane Characterization

#### 3.2.1. Membrane Morphology

**Figure 4:**
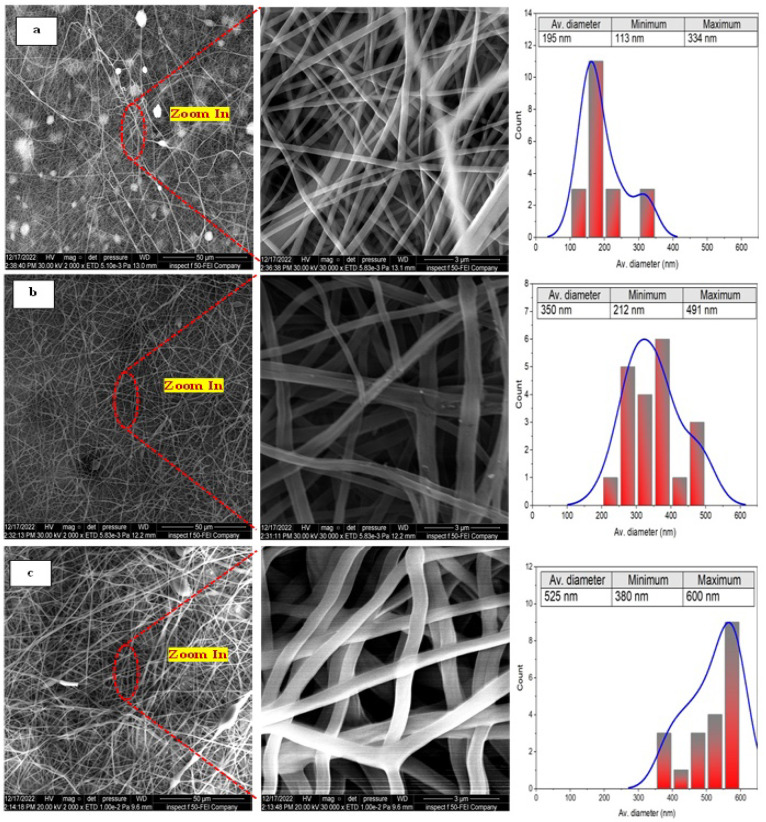
The FE-SEM images of different concentrations of the pristine electrospun PAN nanofiber membranes at different magnifications and the corresponding fiber sizes: (**a**) 10 wt. % PAN/DMF; (**b**) 12 wt. % PAN/DMF; (**c**) 14 wt. % PAN/DMF.

Figure 4 shows the surface morphology and the corresponding fiber size distribution of membranes fabricated at different concentrations of the PAN/DMF precursor solution.

It can be clearly seen that the as-spun PAN nanofibers exhibited randomly oriented 3D nonwoven membranes and an entangled nano-structure with few nanonets for all concentrations of the PAN/DMF precursor solution. Additionally, it can be observed that all the nanofibers did not contain beads, and the average fiber diameter of the membranes was about 195 nm, 350 nm, and 525 nm for 10, 12, and 14 wt. % of the PAN/DMF precursor solution, respectively. The same behavior was noticed by other researchers [56,57,58]. Therefore, the electrospun nonwoven nanofibers’ diameter significantly depends on the concentration of the precursor solution.

**Figure 5:**
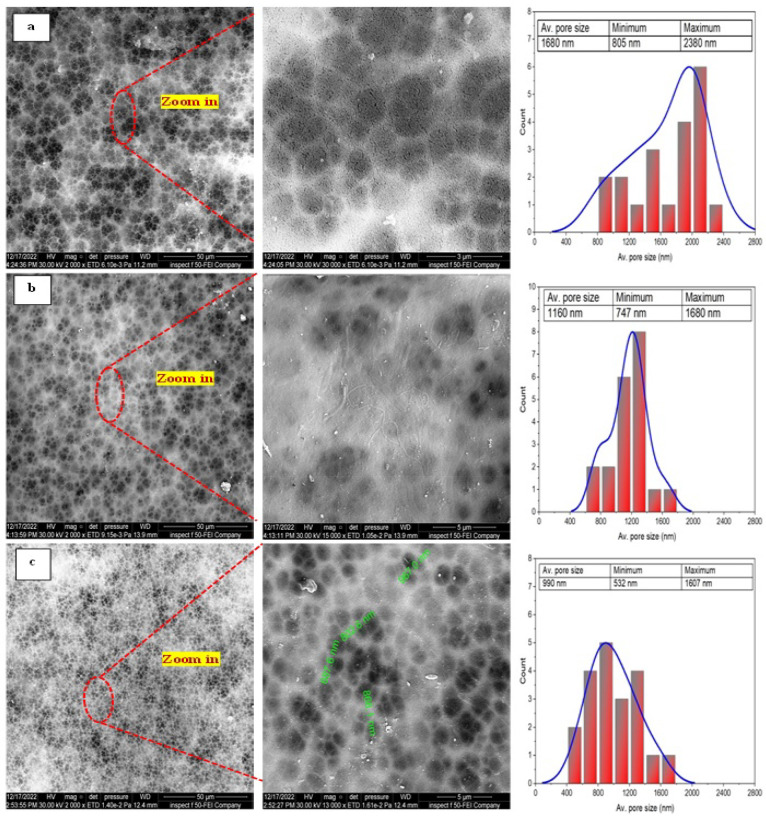
The FE-SEM images of different concentrations of the pristine PAN cast membranes fabricated by phase inversion method at different magnifications and the corresponding pore sizes: (**a**) 10 wt. % PAN/DMF; (**b**) 12 wt. % PAN/DMF; (**c**) 14 wt. % PAN/DMF.

Figure 5 shows the surface morphology and the corresponding pore size of the PAN cast membranes fabricated with different concentrations of PAN/DMF precursor solution prepared by the phase inversion method.

Meanwhile, it can be observed that the size of the macrovoids decreases when the polymer concentration is increased. Therefore, increasing the precursor solution’s PAN concentration makes the membrane denser with a narrower pore size. This behavior is attributed to increasing the solution viscosity, which restricts the movement of polymer chains and causes a reduction in the solvent–non-solvent exchange, resulting in a denser and smoother surface morphology [59].

#### 3.2.2. Membrane Topography

**Figure 6:**
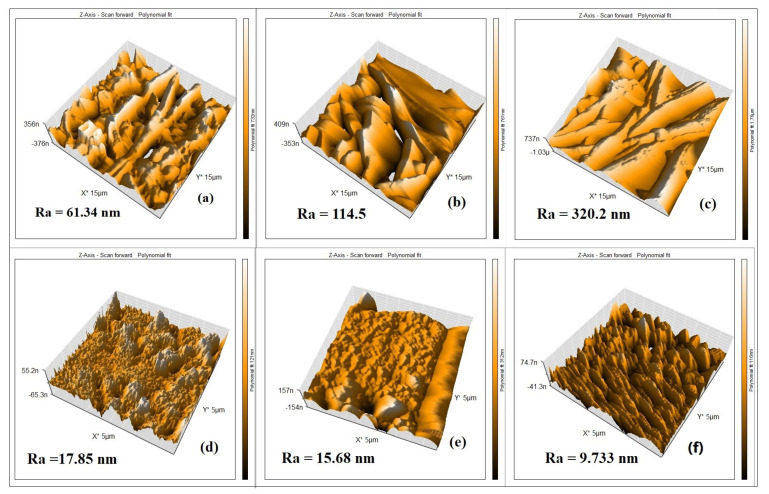
The AFM images of (**a**–**c**) 10, 12, and 14 wt. % PAN electrospun membranes, respectively, and (**d**–**f**) 10, 12 and 14 wt. % PAN cast membranes, respectively.

Figure 6a–c show the AFM images of the 10, 12, and 14 wt. % PAN/DMF electrospun nonwoven nanofibers membranes, respectively. All images were obtained in a (15 µm × 15 µm) frame as a 3D AFM scanning image. These images represent the topography and height of the electrospun nanofibers. It was found that the average surface roughness (Ra) increased from 61 to 320 nm when the precursor solution increased from 10 wt. % to 14 wt. % PAN/DMF membranes. Subsequently, it became clear that the membrane roughness increased when the precursor solution concentrations increased. As mentioned previously, the fiber diameter increases as the concentration of the precursor solution increases. Therefore, the surface roughness of the electrospun membranes is related to the diameter of the nanofibers [60].

Additionally, it is worth mentioning that when comparing the roughness of the 14 wt. % PAN/DMF nanofiber membrane with the 10 and 12 wt. % PAN/DMF electrospun nanofiber membranes, we found that it possesses the highest roughness value. One of the reasons that it has the highest roughness value may be that it includes the highest fiber diameter compared to the other membranes.

Turning to the surface roughness behavior of the cast membranes, Figure 6d–f display the AFM images of three membranes corresponding to (10, 12, and 14) wt. % PAN/DMF. These membranes have an average surface roughness (Ra) of 17.85, 15.68, and 9.73 nm, respectively. Thus, it can be observed that when the polymer concentration is increased, the surface roughness decreases, leading to a smoother surface [59].

When comparing the surface roughness value between the electrospun PAN nanofiber membranes (61–320 nm) and the cast PAN membranes (17–9 nm), it is clear that the membranes produced by the electrospinning process have a higher roughness than the membranes produced by the phase inversion process. Similar findings were reported by Kugarajah in 2021 [61].

#### 3.2.3. Membrane Wettability and Porosity

**Figure 7:**
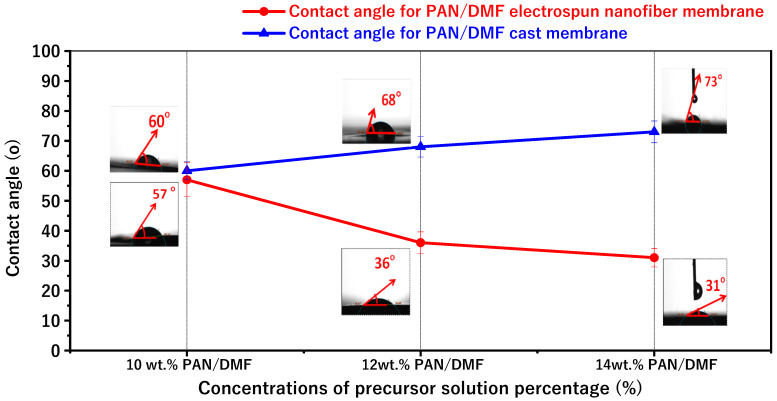
The variations in contact angles and porosity for pristine PAN/DMF electrospinning membrane and PAN/DMF cast membrane at different concentrations of the precursor solution.

The average contact angle values and images for the PAN electrospinning membrane and the PAN cast membrane at different concentrations of the precursor solution at 10, 12, and 14 wt. % PAN/DMF are shown in Figure 7. The contact angles for the electrospun PAN membranes were about 57°, 36°, and 31° for 10, 12, and 14 wt. % PAN/DMF, respectively. Furthermore, the surfaces of all the pristine PAN/DMF electrospinning membranes exhibit hydrophilic behavior.

Additionally, and importantly, there is an opposite relationship between the contact angle and the surface roughness from previous results for the surface roughness. It has been found that the nanofiber membranes with the highest surface roughness value have the lowest water contact angle value. In other words, the higher surface roughness increases the hydrophilicity of the nanofiber surface [62,63].

Similarly, Figure 6 shows the average contact angle for different concentrations of PAN/membrane fabricated by the phase inversion method. The average contact angles were about 60°, 68°, and 73° for 10, 12, and 14 wt. % PAN/DMF, respectively.

According to the above results, the contact angle increases when the concentration of the precursor solution increases. The contact angle was affected by the pore sizes of the membrane: it seems that when the pore sizes of the membrane decrease, the contact angle increases. These results agree with those from Hamta 2021 [64].

Furthermore, when comparing electrospinning and phase inversion, it can be noticed that the electrospun membranes have lower contact angles compared to the cast membranes. So, the electrospinning process produces membranes that are more hydrophilic than those produced by the phase inversion process.

**Figure 8:**
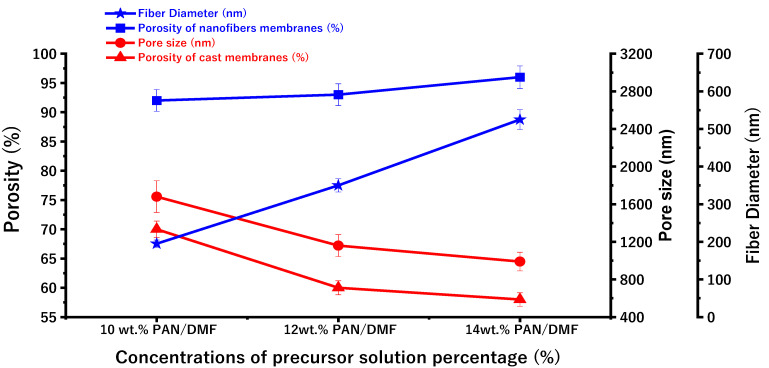
The variations in surface porosity of the PAN/DMF electrospun nanofibers membrane and PAN/DMF cast membrane with fiber diameter and pore size.

Figure 8 shows the porosity of the nanofiber membranes and the cast membranes. The measured porosity of the fabricated electrospun nonwoven nanofiber membranes increased from 92% to 96% when the concentration of the polymeric PAN/DMF solution increased from 10 to 14 wt. %. This can be attributed to the increase in fiber diameter (as shown in Figure 7), which leads to an increase in the macrovoids between the fibers.

The measured porosity of the cast PAN membranes decreased from 70% to 58% when the concentration of the precursor solution increased from 10 to 14 wt. %.

### 3.3. A Comparison between Mechanical Properties of Electrospun Nanofiber Membrane and Cast Membrane

**Figure 9:**
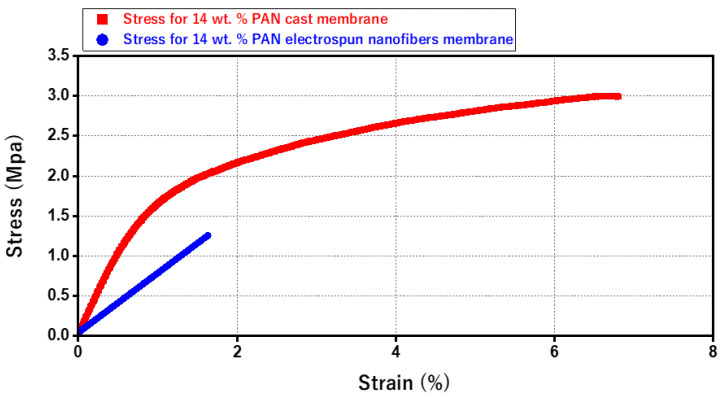
Stress–strain Diagram for the 14 wt. % Electrospun PAN and the 14% wt. % Cast PAN membrane.

Figure 9 illustrates the stress–strain curves of the pristine 14 wt. % PAN/DMF membranes. The electrospun PAN nanofiber membrane reveals a low level of mechanical strength. In contrast, the stress–strain curves of the 14 wt. % PAN cast membrane displays higher mechanical strength and elongation behavior over a longer time. Hence, the cast membrane is stronger and more resilient than the electrospun nanofiber membranes—specifically, the tensile strength of the 14 wt. % PAN cast membrane is 3 MPa, which is approximately 2.4 times higher than the 14 wt. % PAN electrospun nanofiber membrane’s tensile strength of 1.25 MPa.

It is worth mentioning that cast membranes are preferred when high pressure is required in the filtration process, (due to their high mechanical strength). Alternatively, electrospun membranes are highly preferred when working with low or no hydraulic pressure, as in our case.

### 3.4. Performance Results

**Figure 10:**
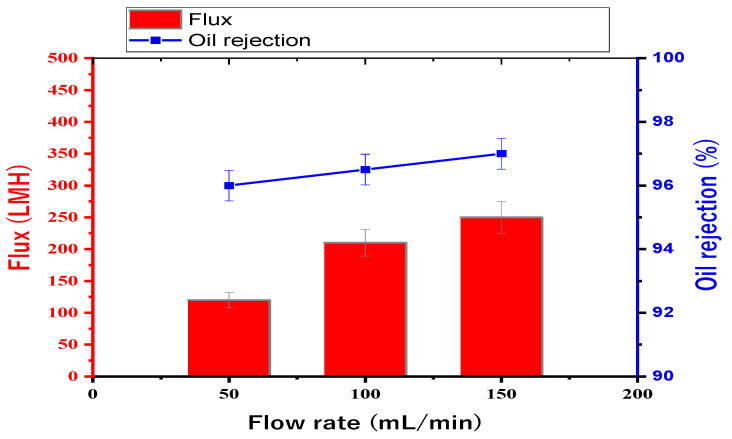
Choosing the optimum feed flow rate from three different feed flow rates: 50, 100, and 150 mL/min.

Figure 10 shows the results of choosing the optimum feed flow rate without any pressure applied at different feed flow rates of 50, 100, and 150 mL/min when testing the 14 wt. % PAN/DMF electrospun nanofibers membrane. The flux and oil rejection percentages were 120, 210, and 250 LMH and 96, 96.5, and 97%, respectively. The flow rate of 150 mL/min was found to have the highest value for both the flux and oil rejection. Therefore, all membranes were tested at the optimum feed flow rate of 150 mL/min.

**Figure 11:**
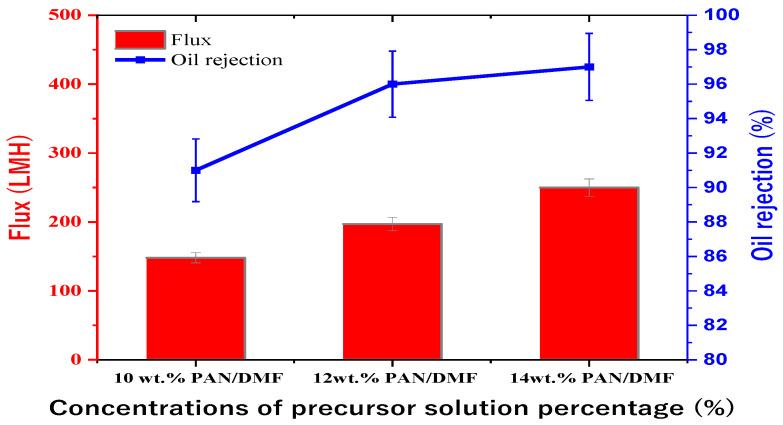
Performance of PAN/DMF electrospun nanofiber membranes at different concentrations of precursor solution.

**Figure 12:**
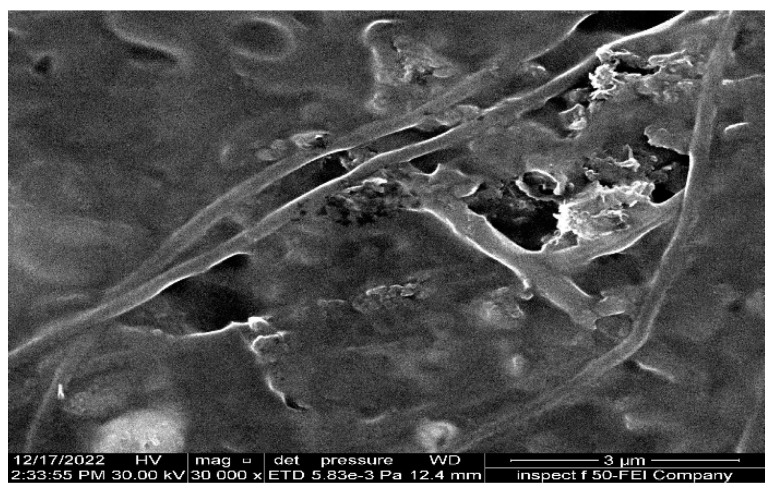
SEM images of fouling for 10 wt. % PAN-based electrospun nanofiber membrane after undergoing an emulsified oil separation test.

The study investigated the separation performance of PAN electrospun membranes with different concentrations of precursor solutions (i.e., 10, 12, and 14 wt. % PAN/DMF), as illustrated in Figure 11. All PAN/DMF membranes exhibited an oil rejection of over 91%, with the 14% PAN membrane showing the highest oil rejection of 97%. Furthermore, the 14 wt. % PAN electrospun nanofiber membrane demonstrated the highest flux of 250 LMH among the tested membranes. This can be attributed to its higher surface hydrophilicity and porosity, as discussed earlier [65]. On the other hand, the 10 wt. % PAN/DMF electrospun nanofiber membrane exhibited the lowest performance due to its low surface hydrophilicity and porosity. This led to fouling accumulation on the membrane surface, plugging all pores, as shown in Figure 12, and negatively impacted its performance in terms of both flux and oil rejection.

**Figure 13:**
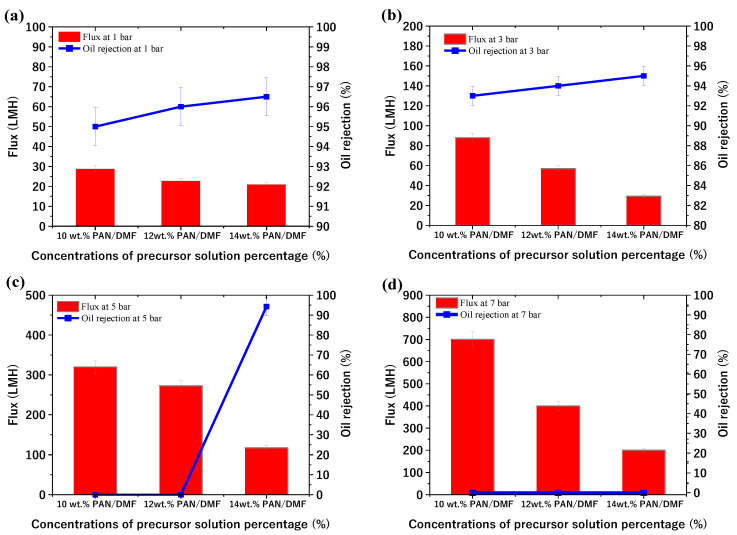
Performance of PAN membranes fabricated by phase inversion method at different concentrations of precursor solutions and different applied pressures at (**a**) 1 bar, (**b**) 3 bar, (**c**) 5 bar, and (**d**) 7 bar.

The study also investigated the performance of the PAN membranes fabricated by the phase inversion method at different concentrations of precursor solution and different applied pressures (1, 3, 5, and 7 bar). The membrane performance (flux and oil rejection) at 1 bar was 28.8, 22.8, and 21 LMH and 95, 96, and 96.5% for the 10, 12, and 14% PAN/DMF cast membranes, respectively. Additionally, the flux increased at a pressure of 3 bar, while the oil rejection slightly decreased. It is worth noting that the 10 and 12 PAN/DMF cast membranes were ruptured at the beginning of the test at 5 and 7 bar, as shown in Figure 13c,d. Additionally, no cast membranes (including the 14% PAN/DMF membrane) were tested at a pressure higher than 7 bar as this caused the membranes to rupture. This is due to the limited mechanical strength of the membrane. However, the 14 wt. % PAN/DMF cast membrane at 5 bar gave the best separation performance, in which the flux and oil rejection were 117 LMH and 94.3%, respectively.

**Figure 14:**
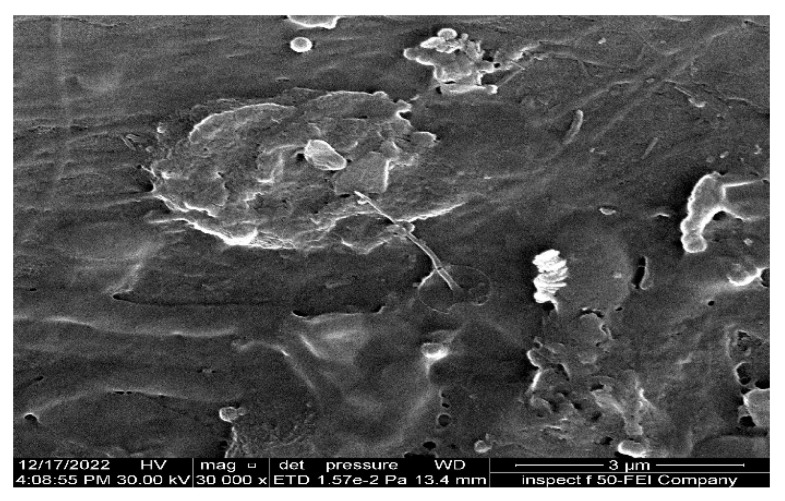
SEM images of fouling for 14 wt. % PAN cast membrane after undergoing an emulsified oil separation test.

Additionally, Figure 14 illustrates the fouling on the surface of the 14 wt. % PAN cast membrane. This occurred because it has the smallest pore size compared to the other membranes, which prevents oil droplets from passing through it. As a result, fouling accumulates on the membrane surface, and a cake layer is formed.

It was also noticed that the flux of the PAN/DMF cast membranes decreased when the polymer concentration increased. There is a positive indirect relationship between the pore size and the flux of the cast membranes [66]. As shown in the previous sections, the cast membranes’ pore size decreases when the precursor concentration increases.

When comparing the separation performance between the 14% PAN/DMF nanofiber membrane with the 14% PAN/DMF cast membrane at a pressure of 5 bar, it can be observed that the nanofiber membrane showed a significantly better separation performance with the great advantage of not requiring a higher applied pressure. Therefore, in this case, this advantage of the electrospinning process without using any applied pressure will make this process more economical because a higher applied pressure leads to a higher operating cost [67].

### 3.5. Evaluation of the Antifouling of the Membrane Performance

When fouling adheres to a membrane, it can cause a series of troubles, such as blocking pores, decreasing the efficiency, shortening the life of the membrane, etc. [68].

**Figure 15:**
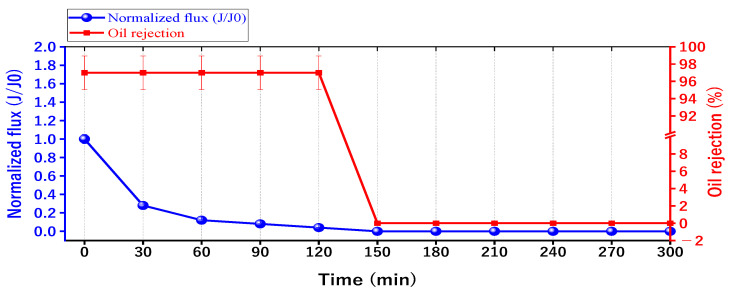
The separation performance with the time for the 14 wt. % PAN/DMF electrospun nanofiber membrane.

The optimum membrane of 14% PAN/DMF electrospun membrane was chosen to study the effect of oil fouling on the membrane performance, as shown in Figure 15. The results revealed that after 150 min of continuous operation, the membrane was completely blocked without any flux. It is worth mentioning that the oil rejection was slightly affected, decreasing from 97% to 96.5%. The reason for the decline in the performance of the membrane is due to its continuous operation over a prolonged period [69], which results in the formation of a cake layer on the membrane surface [25]. This cake layer accumulates a significant amount of oil particles, leading to fouling and the complete coverage of the membrane surface. As a result, low porosity is obtained, and a high specific cake resistance is observed [70]. Moreover, the continuous accumulation of fouling leads to complete pore blockage in the membrane, as shown in Figure 12. For this reason, the periodic cleaning (backwashing) of the membrane for regeneration was required to remove the accumulated fouling.

**Figure 16:**
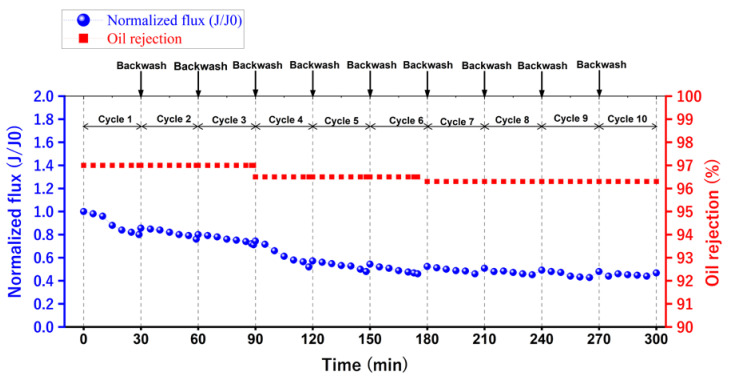
The separation performance for ten repeated cycles for the optimum 14 wt. % PAN/DMF electrospun nanofiber membrane.

To evaluate the membrane’s performance, it was necessary to overcome this fouling. Therefore, the membrane was back washed with distilled water after every cycle for 5 min. So, Figure 16 shows the separation performance for ten repeated cycles. Additionally, it is obvious that after ten cycles of separation, the membrane maintained an acceptable separation performance for emulsified oil.

A comparison of the pristine 14 wt. % PAN/DMF nanofiber membrane with some of the previously reported electrospun PAN membranes tested in oil removal can be found in Table 1. The table also highlights the differences in filtration methods employed in these studies. Notably, no additives were used to enhance flux and oil rejection in this study, but this could be a topic for future research. Our membrane showed higher water flux and comparable oil rejection compared to the previous research that utilized cross-flow systems.

In dead-end filtration, the flow direction is perpendicular to the membrane surface, leading to the accumulation of larger particles on the surface and forming a cake that may block the channels. In contrast, cross-flow filtration involves the flow direction running parallel to the membrane surface at high velocity, which sweeps away the particles from the surface [71]. Cross-flow ultrafiltration is more commonly used in commercial and industrial applications because dead-end filtration has limitations, such as a short fouling time and the need for frequent cleaning. Cross-flow ultrafiltration offers better control over various parameters, such as filtration/backwashing cycles, filtration surface area, and filtration flux, making it a more practical and cost-effective option for industrial applications [72].

**Table 1:** Summary of the performance of different PAN nanofiber membranes selected from the literature.

Materials	Solvent	Concentration of Precursor Solution	Flux (LMH)	Oil Rejection (%)	Filtration Method	Ref.
PAN/PVA	(DMF) and water	(8 wt. % PAN/DMF) deposited on (8 wt. % PVA/water)	210	99.5	Cross-flow filtration (at feeding pressure of 0.3 MPa)	[73]
PAN@ZIF-8	(DMF) and methanol	(10 wt. %PAN/16 wt. % ZIF-8)/DMF	>900	>99.95	Dead-end filtration	[74]
PAN PAN/HPEI PAN/HPEI/PDA	(DMF)	Pristine 17 wt. % PAN/DMF	800	<98	Dead-end filtration	[75]
PAN PAN/PANI	(DMF)	- Pristine 8 wt. % PAN/DMF - PAN/PANI (coated)	325 290	90.2 99.8	Dead-end filtration	[76]
PAN PAN/PS	(DMF) and (LiCl)	Pristine 14 wt. % PAN/DMF (single layer)	258	-	Dead-end filtration	[77]
Au@ZIF-8@PAN-TD	(DMF), methanol, and water	(16 wt. % PAN + 0.1 wt. % f Au@ZIF-8 NPs)/DMF	>200	97.8	Dead-end filtration	[78]
PAN/PVP	(DMF)	Crosslinking 10 wt. % PVP/PAN	569	-	Dead-end filtration	[68]
CNTs-PAN	(DMF)	CNTs-PAN [(8 + 5) wt. % PAN/DMF (double layer heat-pressed) + (0.2 wt% CNTs and 0.05 wt% PVA) and 0.05 wt% Glutaraldehyde (GA)]	60	96	Cross-flow filtration (at feeding pressure of 0.3 MPa and an applied pressure of 0.02 MPa)	[79]
PAN PAN/PMMA	(DMF) and (ACE)	Pristine 11 wt. % PAN/DMF	120	96	Cross-flow filtration (without pressure)	[51]
PAN	(DMF)	Pristine 14 wt. % PAN/DMF	250	97	Cross-flow filtration (without pressure)	This work

## 4. Conclusions

This study compared two membrane fabrication techniques, electrospinning and phase inversion, at varying concentrations (10, 12, and 14 wt. %). The concentration of the precursor solution plays a crucial role in determining the diameter of the nanofibers in electrospun nonwoven membranes and the pore size in cast membranes. Moreover, it leads to an increase in roughness for both types of membranes. Furthermore, the increase in precursor concentration enhanced the hydrophilicity for the electrospun nanofiber membranes, while it caused a decrease in hydrophilicity for the cast membranes. The same trend is observed for the porosity, for which the electrospun nanofiber membrane showed an increase, while the cast membrane exhibited a decrease. In terms of performance, the electrospun PAN nanofiber membranes displayed more flux at 250 LMH and more oil rejection at 97% compared to the cast PAN membranes. The reusability of the electrospun PAN membrane was studied for up to 10 cycles. It showed an acceptable separation performance for oil rejection and water flux with the advantage of not using applied pressure.

Therefore, the electrospinning process without using any applied pressure will make this process more economical. Furthermore, the unique characteristics of electrospun nanofiber membranes make them promising and potentially essential for industrial applications, particularly for treating oily wastewater. However, large-scale electrospun membrane production and testing on a long-scale plant are highly recommended for future work.

## Data Availability

Not applicable.
